# How Consistent Are Challenge and Threat Evaluations? A Generalizability Analysis

**DOI:** 10.3389/fpsyg.2019.01778

**Published:** 2019-08-02

**Authors:** Lee J. Moore, Paul Freeman, Adrian Hase, Emma Solomon-Moore, Rachel Arnold

**Affiliations:** ^1^Department for Health, University of Bath, Bath, United Kingdom; ^2^School of Sport, Rehabilitation, and Exercise Sciences, University of Essex, Colchester, United Kingdom

**Keywords:** cognitive appraisals, demand and resource evaluations, generalizability theory, roller derby, stressors, stress appraisals, stress management, variance partitioning approaches

## Abstract

Viewing stressful situations as more of a challenge than a threat (i.e., coping resources match or exceed situational demands) has been associated with better performance and long-term health. However, to date, little research has examined if individuals have tendencies to evaluate all stressful situations as more of a challenge or threat. Thus, this study used generalizability analyses to investigate the consistency (or variability) of challenge and threat evaluations across potentially stressful situations. 1813 roller derby players (89.0% female; *M*_age_ = 33 years, *SD* = 7) read nine stressful vignettes (e.g., injury, non-selection, family illness), before completing self-report items assessing challenge and threat evaluations. Generalizability analyses revealed that the Athlete × Stressor interaction accounted for the greatest amount of variance in challenge and threat evaluations (51.9%), suggesting that athletes had idiosyncrasies in their tendency to view particular stressors as more of a challenge or threat. The Athlete (15.4%) and Stressor (21.9%) components also accounted for a significant amount of variance. While the Athlete component suggested some consistency in challenge and threat evaluations, and that differences existed between athletes in whether they tended to view stressors as more of a challenge or threat, the Stressor component indicated some agreement among the athletes in their tendency to view some stressors as more of a challenge or threat than others. The findings offer direct support for transactional stress theories, and have important implications for practitioners developing stress management interventions.

## Introduction

Sport is inherently stressful, with athletes required to cope with the multiple demands they face during competition (e.g., high pressure), day-to-day training (e.g., coach conflict), and their personal lives (e.g., family duties; Fletcher et al., 2006). The ability to cope with stress is highly sought-after, and is a psychological skill that characterizes world-class athletes (e.g., Olympians; Gould et al., 2002). As such, researchers continue to develop interventions that help athletes manage stress (e.g., mindfulness; see Rumbold et al., 2012; Randall et al., 2019 for reviews). To aid intervention development, researchers have tested models that explain how athletes evaluate stressful situations, and whether these evaluations, and subsequent responses, vary between athletes and across situations (e.g., model for coping with acute stress in sports; Anshel, 2001). One theory that has gained recent attention, and is the focus of this study, is the biopsychosocial model (BPSM) of challenge and threat states (Blascovich, 2008a).

Akin to other transactional stress theories (e.g., cognitive appraisal theory; Lazarus and Folkman, 1984), the BPSM states that when faced with a stressful situation, an athlete evaluates the demands of the situation and whether they possess the resources to cope with those demands (Blascovich, 2008a). If an athlete perceives that they have sufficient resources, they evaluate the situation as a challenge. However, if an athlete perceives that they lack the resources, they evaluate the situation as a threat (Blascovich, 2008a). The BPSM argues that these evaluations trigger distinct physiological responses (Seery, 2011). Specifically, inspired by the theory of physiological toughness (Dienstbier, 1989), a challenge evaluation initiates sympathetic-adrenomedullary activation and the release of catecholamines (e.g., adrenaline), resulting in dilation of the blood vessels and increased blood flow (marked by reduced total peripheral resistance and elevated cardiac output). Conversely, a threat evaluation triggers pituitary-adrenocortical activation and the release of cortisol, causing little change or constriction of the blood vessels and little change or decreased blood flow (marked by little change or elevated total peripheral resistance and little change or reduced cardiac output; Seery, 2011). Despite their discrete labels, challenge and threat are not conceptualized as dichotomous states but anchors of a bipolar continuum, meaning that relative rather than absolute differences in challenge and threat are often examined (e.g., situation evaluated as more or less of a challenge or threat; Seery, 2011).

The BPSM posits that a challenge state should lead to better performance than a threat state (Blascovich, 2008a). Research has supported this assertion (see Behnke and Kaczmarek, 2018; Hase et al., 2018 for reviews), demonstrating that athletes perform more optimally when they evaluate situations as more of a challenge (resources match or exceed demands; e.g., Moore et al., 2013), and respond to situations with cardiovascular reactivity more consistent with a challenge state (reduced total peripheral resistance and elevated cardiac output; e.g., Turner et al., 2013). Beyond their short-term effects on performance, challenge and threat states are also thought to impact long-term health (Blascovich, 2008b). Indeed, repeatedly evaluating stressful situations as a threat has been linked with poor mental health (e.g., depression), and frequently responding to situations with more threat-like cardiovascular reactivity has been associated with heart disease (Blascovich, 2008b). Despite these important outcomes, little research has explored the consistency (or variability) of challenge and threat, and whether individuals have tendencies to evaluate all stressful situations as more of a challenge or threat. This is surprising given that psychometric tools assessing individual differences in challenge and threat have recently emerged (Tomaka et al., 2018), and that while limited, evidence has hinted that threat evaluations are moderately to highly consistent across situations (e.g., Power and Hill, 2010).

One approach that could help elucidate the consistency (or variability) of challenge and threat evaluations is generalizability theory (Cronbach et al., 1972). Generalizability theory is a variance partitioning approach that is used to examine within-person variation, and specifically person × situation interactions, or differences between individuals in their perceptions and responses across the same situations (see Lakey, 2016 for a review). Generalizability theory has been applied to a range of psychosocial constructs to understand if these constructs are features of the person, situation, or person × situation interactions (e.g., social support; Lakey, 2010). For instance, Endler and Hunt (1966, 1969) applied generalizability theory to anxiety, asking participants to rate their anxiety in response to various situations (e.g., giving a speech, long car drive). Person effects accounted for 8% of variance in anxiety, suggesting some participants reported more anxiety than others across the situations. In addition, situation effects accounted for 7% of variance, implying that some situations evoked more anxiety than others, across all participants. Finally, person × situation interactions accounted for 17% of variance, suggesting idiosyncrasies in anxiety responses, and that participants reported different levels of anxiety across situations (e.g., some participants rated the speech as more anxiety-provoking than the long car drive, while others rated the drive as more anxiety-provoking). Thus, both person and situation effects explained small but meaningful proportions of variance in anxiety, and their interaction represented the largest variance component, explaining twice as much variance as the individual components. Despite its potential to improve our understanding of stress responses, generalizability theory has rarely been applied to this psychosocial construct, possibly due to the conceptual and analytical complexities associated with this approach (Lakey, 2016).

To the authors’ knowledge, to date, only one study has used generalizability theory in the stress literature (Lucas et al., 2012). Lucas and colleagues found that the stress appraisals of police officers were primarily comprised of person × situation interactions (38–41% of variance), although the person and situation effects were also significant (14–15 and 18–19% of variance, respectively). The findings offered direct support for transactional theories (e.g., cognitive appraisal theory; Lazarus and Folkman, 1984), which conceptualize stress as a psychosocial construct that emerges from interactions between the individual and their environment. Furthermore, the findings had implications for stress management interventions, highlighting that to be effective, such interventions should move beyond solely individual- or environment-based approaches, and instead take a conjoint approach that considers who is encountering what particular stressors (e.g., cognitive-behavioral strategies that allow individuals to acquire new skills that they can use to cope with the stressors that they find uniquely stressful; Giga et al., 2003). Therefore, by illuminating the relative importance of different sources of variance in stress responses (person, situation, or person × situation effects), generalizability analyses can offer a direct test of theory and have important implications for the creation of stress management interventions.

Using generalizability theory, this study investigated the consistency (or variability) of challenge and threat evaluations across potentially stressful situations. Although the athlete (person) and stressor (situation) effects were expected to be significant, with a greater athlete effect suggesting that challenge and threat evaluations were relatively consistent across stressful situations, it was predicted that the athlete × stressor (person × situation) interaction effect would also be significant and account for the greatest amount of variance in challenge and threat evaluations. This interaction effect would reflect unique matches between athletes and stressors, or idiosyncrasies in the tendency for athletes to view certain stressors as more of a challenge or threat. The same pattern of significant effects were also expected when demand and resource evaluations were examined separately.

## Methods

### Participants

Roller derby players were recruited via advertisements and a link to the study posted on publicly available internet message boards (e.g., Facebook), and by emailing teams and asking them to share the link. In total, the link was opened 2628 times, with 2176 participants partially completing the survey, however, 363 were missing challenge and threat evaluation data for all stressful vignettes. Thus, the final sample consisted of 1813 participants (140 males, 1625 females, 48 preferred to self-describe; demographic and sport-specific characteristics are summarized in Table 1). Participants were aged between 18 and 78 years (*M*_age_ = 33 years, *SD* = 7), and had been playing roller derby for between 0 (less than 1 year) and 14 years (*M*_experience_ = 4 years, *SD* = 2). All participants provided written informed consent in line with the Declaration of Helsinki.

**Table 1:** Demographic and sport-specific characteristics of the participants (*n* = 1813).

	*n*	%
**Gender**		
Male	140	7.7
Female	1625	89.6
Preferred to self-describe	48	2.7
**Nationality**		
European	902	49.8
North American	787	43.4
Australian	81	4.5
Other (South American, Asian etc.)	15	0.8
Did not report	28	1.5
**Competitive level**		
International	155	8.6
Advanced	577	31.8
Intermediate	758	41.8
Rookie	322	17.8
Did not report	1	0.1

### Procedure and Measures

Following institutional ethical approval, an online survey was created using Qualtrics software. The survey took ∼15 min to complete. In the first part, participants reported demographic and sport-specific information (age, gender, nationality, competitive level, and playing experience). In the second part, participants read nine vignettes, each describing a potentially stressful situation (e.g., ‘deselection,’ ‘family illness’; see section “Stressful Vignettes”). After reading each vignette, participants completed four self-report items, two from the cognitive appraisal ratio (CAR; Tomaka et al., 1993), and two from the stressor appraisal scale (SAS; Schneider, 2008). Specifically, to assess evaluations of situational demands in response to each vignette, participants were asked “how demanding would you find this situation?” (CAR), and “how stressful would you find this situation?” (SAS). Furthermore, to assess evaluations of coping resources, participants were asked “how well would you be able to cope with the demands of this situation?” (CAR), and “how well do you think you could manage the demands imposed on you in this situation?” (SAS). All items were rated on six-point Likert scales anchored between 1 (*not at all*) and 6 (*extremely*). The items were then converted into two demand resource evaluation scores (DRES). The first DRES score, termed DRES-CAR, was calculated by subtracting the first demands item (“how demanding would you find this situation?”) from the first resources item (“how well would you be able to cope with the demands of this situation?”). The second DRES score, labeled DRES-SAS, was calculated by subtracting the second demands item (“how stressful would you find this situation?”) from the second resources item (“how well do you think you could manage the demands imposed on you in this situation?”). Both DRES scores ranged from −5 to +5, with positive values reflecting challenge evaluations (resources match or exceed demands), and negative values reflecting threat evaluations (demands exceed resources; as Moore et al., 2018).

### Stressful Vignettes

Inspired by research highlighting the stressors commonly experienced in sport (e.g., Arnold and Fletcher, 2012a; Sarkar and Fletcher, 2014), two subsets of potentially stressful vignettes were created (as Lucas et al., 2012). Two separate subsets were used to reduce the length of the survey (36 vs. 72 items), and thus improve completion rates and sample size. Each subset contained nine vignettes, with three describing competitive stressors (e.g., ‘underperforming’), three outlining organizational stressors (e.g., ‘travel’), and three describing personal stressors (e.g., ‘relationship problems’). A diverse set of stressors was selected to offer a better test of the consistency (or variability) of challenge and threat evaluations compared to a more uniform set of stressors (e.g., competitive only). All stressors were processive rather than systemic (i.e., required cognitive processing vs. purely physiological in nature; Anisman, 2014). Participants were randomly assigned to one of the two subsets by the Qualtrics survey, and the order in which the vignettes were presented within each subset was also randomized. The content and wording of each vignette was developed by the lead researcher and edited to improve sport-specificity by another member of the research team who was an experienced roller derby coach. Additionally, each vignette was scrutinized by two other coaches to ensure that the content was relevant, and the length, tone, and focus were appropriate. Each vignette is presented in full in Table 2.

**Table 2:** The potentially stressful vignettes (or stressors) used in the study, grouped by subset.

Subset 1	
Inadequate preparation (C)	Due to factors outside of your control, you have arrived at the venue with only 20 min until the start of your game and first whistle…you feel under prepared as you have had no time to hydrate, warm-up, or test the floor…to make matters worse, you missed the team talk where the coach/captain talked tactics…
Injury (C)	You are about to play against a team known for being particularly aggressive and deliberately trying to injure their opponents…when you last played this team, one of your teammates suffered a serious injury, breaking their ankle…you know that if this happens to you, you will be unable to work…
Outcome Pressure (C)	It is moments until the last game of your competitive season and you are in the final of a roller derby tournament…if you win, you will be crowned champions, lift the trophy, and climb the rankings…however, if you lose, you will have failed, watch your opponents lift the trophy, and drop in the rankings…
Coach’s personality and behavior (O)	A new coach has just joined your roller derby team and is now your bench manager during games…you do not like their personality, you think they are arrogant and get too angry…you are warming-up before an important game and the new coach is shouting at the team, accusing you and your teammates of being lazy…
Officials (O)	It is 15 min into the second half of a ‘must-win’ game…in the middle of a fiercely contested and intense jam, you hear a whistle from the referee who calls your number and gives you a penalty…when skating off, you realize that the referee has wrongly sent you to the penalty box, calling a ‘cutting’ penalty against you…
Spectators (O)	You are about to start a jam midway through the first half of an away game…there is a large and raucous crowd watching, most of which are supporting your opponents…the crowd are chanting loudly for your opponents, jeering at you and your teammates, calling penalties against you, and hassling the referees…
Balancing training and work (P)	You have been struggling to juggle work and derby recently…work has become harder and you have had to work more hours, and as a result you have been unable to train and practice your skills…it is now moments before the first whistle of an important game for your roller derby team…
Missing friends and family (P)	You are homesick, and missing your close friends and family…you have been away at a roller derby tournament which is several hours from home, and have been staying in a hotel with teammates the last two nights…you are now stood on the track waiting for the final game of the long, 2-day, tournament to begin…
Relationship problems (P)	Your family normally watch all of your roller derby games, home or away…however, you had a big argument with a family member last week and you have not spoken to them since, and they have not come to support you today…you have just warmed-up and your important game is about to start…
**Subset 2**	
Underperforming (C)	It is half-time in an important game and you are not playing very well…you have already had six visits to the penalty box and you know that if you are given one more penalty you will foul out of the game…you feel like you have let yourself and your teammates down with your awful performance…
Expectations (C)	You are just about to play against a team ranked far lower than you in the rankings…you are expecting your team to win by a huge margin and know you need to do so in order to move up in the rankings…personally, you are expecting to put in a brilliant performance and win one of the individual awards…
Self-presentation (C)	You have been selected to play for your roller derby team in an important game…just before the game is about to start, your coach/captain tells you that they will be assessing your skills and evaluating your performance…the coach/captain is considering promoting you to a higher team, rotation, or position…
Teammate attitude (O)	You have one teammate who thinks they are superior to you and has a bad attitude…you are returning to the bench after making a mistake which resulted in your team losing the last jam…when you get to the bench, this teammate shouts at you in front of the team, telling you how bad you are and what you need to do…
Selection (O)	Your team have qualified for an important roller derby tournament…you have attended every training session recently and have been playing the best derby since you started skating…just before the first game of this tournament, your coach tells you that you have been selected as reserve, and you will play very few jams…
Travel (O)	You have finally arrived at the venue of an important game your team cannot afford to lose…you had to wake-up very early to catch the team bus and then spent hours on the bus fighting through heavy traffic to get to the venue…on the bus, you had to sit next to a new teammate you have hardly spoken to before…
Family illness (P)	Your parents often come to watch and support you during your roller derby games, home or away…however, one of your parents has recently been diagnosed with a serious illness and so they are not there to watch you today…it is now seconds before the final game of the season and a game your team must win…
Death of a friend (P)	You have had an emotional and difficult few weeks leading up to an important game for your roller derby team…unfortunately, one of your best friends died recently in a sudden and tragic accident…you are now on the track moments before the first whistle of a crucial game for your roller derby team…
Financial issues (P)	The last couple of months have been challenging and difficult for you…a number of unexpected bills have left you struggling with your finances recently, plummeting you into large amounts of debt…you are now warming-up and practicing your skills before the final game of a large roller derby tournament…

*C, competitive stressor; O, organizational stressor; P, personal stressor.*

### Statistical Analyses

Consistent with previous generalizability theory research (e.g., Lakey et al., 2010; Lucas et al., 2012), variance components analyses with restricted maximum likelihood estimation were conducted separately for DRES, demand evaluations, and resource evaluations in IBM SPSS statistics software (version 22). For each outcome, the analysis had an 1813 (Athletes) × 18 (Stressors) × 2 (Items) × 2 (Subsets) design. Stressors and Items were within-participants factors, and Athletes and Subsets were between-participants factors. However, because the design was not fully crossed (i.e., Stressors and Athletes nested within Subsets), estimates of variance related to Stressors were adjusted accordingly (by specifying nested terms; e.g., Stressors [Subsets] and Athletes [Subsets]). The highest order interaction term was confounded with error and variance not attributable to any measured effect or component (Shavelson and Webb, 1991). The Stressor [Subsets], Athlete [Subsets], and the Athlete × Stressor were the key components of interest, but others were also estimated (Items, Subsets, Item × Stressor, Item × Athlete, Item × Subset, and Athlete × Subset). The significance of all estimated sources of variance was examined using 95% confidence intervals, where significant sources did not include or cross zero. The components were considered significantly different from one another if their 95% confidence intervals did not overlap (Field, 2013). Each raw variance component was converted into a percentage of total variance to provide a more meaningful measure of effect size.

## Results

### Descriptive Statistics

The descriptive DRES, demand evaluation, and resource evaluation data for each vignette are presented in Table 3, grouped by subset. On average, participants evaluated the vignettes entitled ‘outcome pressure,’ ‘officials,’ ‘spectators,’ ‘missing friends and family,’ ‘relationship problems,’ ‘expectations,’ ‘selection,’ ‘travel,’ and ‘financial issues’ as more of a challenge (resources match or exceed demands). Conversely, participants evaluated the vignettes entitled ‘inadequate preparation,’ ‘injury,’ ‘coach’s personality and behavior,’ ‘balancing training and work,’ ‘underperforming,’ ‘self-presentation,’ ‘teammate attitude,’ ‘family illness,’ and ‘death of a friend’ as more of a threat (demands exceed resources). Indeed, ‘teammate attitude’ and ‘underperforming’ were evaluated as most demanding, whereas ‘missing friends and family’ and ‘travel’ were rated as least demanding. Moreover, participants evaluated that they were most able to cope with ‘missing friends and family’ and ‘expectations,’ but least able to cope with ‘teammate attitude’ and ‘death of a friend.’

**Table 3:** Mean (SD) demand resource evaluation score (DRES), demand evaluation, and resource evaluation data for the different potentially stressful vignettes (or stressors), grouped by subset (*n* = 1813).

	DRES (−5 to +5)		Demands (1 to 6)		Resources (1 to 6)	
	DRES-CAR	DRES-SAS	CAR	SAS	CAR	SAS
**Subset 1 (*n* = 919)**						
Inadequate preparation (C)	−0.81 (2.06)	−0.93 (2.04)	4.60 (1.27)	4.70 (1.28)	3.79 (1.11)	3.77 (1.10)
Injury (C)	−0.41 (2.13)	−0.41 (2.17)	4.35 (1.35)	4.34 (1.37)	3.93 (1.14)	3.93 (1.14)
Outcome pressure (C)	0.03 (1.78)	0.28 (1.86)	4.38 (1.35)	4.09 (1.33)	4.40 (0.95)	4.36 (0.95)
Coach’s personality and behavior (O)	−1.21 (2.21)	−1.24 (2.21)	4.64 (1.33)	4.65 (1.34)	3.43 (1.26)	3.41 (1.24)
Officials (O)	1.21 (2.20)	1.06 (2.26)	3.32 (1.44)	3.42 (1.45)	4.54 (1.12)	4.48 (1.19)
Spectators (O)	1.12 (2.25)	1.20 (2.22)	3.35 (1.50)	3.22 (1.46)	4.46 (1.09)	4.42 (1.10)
Balancing training and work (P)	−0.19 (1.82)	−0.05 (1.92)	4.14 (1.23)	3.96 (1.27)	3.94 (1.00)	3.91 (0.99)
Missing friends and family (P)	2.17 (2.13)	2.50 (1.94)	2.73 (1.48)	2.35 (1.24)	4.90 (0.95)	4.86 (0.99)
Relationship problems (P)	1.35 (2.35)	1.29 (2.34)	3.11 (1.48)	3.15 (1.49)	4.46 (1.16)	4.43 (1.15)
Subset 1 Mean	0.36 (2.10)	0.41 (2.11)	3.84 (1.38)	3.77 (1.36)	4.21 (1.09)	4.17 (1.09)
**Subset 2 (*n* = 894)**						
Underperforming (C)	−1.26 (1.85)	−1.26 (1.90)	4.78 (1.13)	4.73 (1.16)	3.52 (1.13)	3.47 (1.11)
Expectations (C)	1.52 (2.06)	1.83 (2.00)	3.20 (1.46)	2.83 (1.33)	4.73 (0.97)	4.67 (1.01)
Self-presentation (C)	−0.12 (2.06)	0.04 (2.17)	4.32 (1.38)	4.13 (1.42)	4.20 (1.09)	4.17 (1.10)
Teammate attitude (O)	−1.41 (2.41)	−1.48 (2.45)	4.73 (1.41)	4.84 (1.36)	3.32 (1.43)	3.35 (1.45)
Selection (O)	0.59 (2.50)	0.49 (2.50)	3.42 (1.60)	3.58 (1.60)	4.00 (1.34)	4.07 (1.33)
Travel (O)	1.77 (2.41)	1.71 (2.38)	2.89 (1.56)	2.90 (1.55)	4.66 (1.13)	4.61 (1.13)
Family illness (P)	−0.08 (2.40)	0.06 (2.39)	4.02 (1.50)	3.92 (1.51)	3.95 (1.28)	3.98 (1.24)
Death of a friend (P)	−1.36 (2.40)	−1.08 (2.47)	4.69 (1.37)	4.43 (1.47)	3.33 (1.39)	3.35 (1.37)
Financial issues (P)	0.89 (2.20)	0.79 (2.25)	3.42 (1.47)	3.50 (1.51)	4.31 (1.11)	4.30 (1.08)
Subset 2 Mean	0.06 (2.26)	0.12 (2.28)	3.94 (1.43)	3.87 (1.43)	4.00 (1.21)	4.00 (1.20)

*C, competitive stressor; O, organizational stressor; P, personal stressor; CAR, cognitive appraisal ratio; SAS, stressor appraisal scale.*

### Demand Resource Evaluation Score

The percentages and significance of variance components for DRES are presented in Table 4. The Athlete × Stressor component (interaction effect) accounted for the greatest amount of variance in DRES (51.9%), suggesting that athletes had different profiles of challenge and threat evaluations across the same stressors. This interaction component accounted for a significantly greater amount of variance in DRES than the Athlete (15.4%) and Stressor (21.9%) components, although these were also significant. The Athlete component (person effect) implied that the athletes differed in whether they tended to view the stressors as more of a challenge or threat, regardless of the characteristics of the stressors. The Stressor component (situation effect) suggested some agreement among the athletes in their tendency to view some stressors as more of a challenge or threat than others. The variance attributable to the Athlete and Stressor components did not differ significantly.

**Table 4:** Percentages of variance and significance of DRES, demand evaluations, and resource evaluations.

Source	σ^2^	% σ^2^	95% CI
**DRES**			
Subset	0.00	0.00	N/A
Stressor (Subset)	1.36	21.89	(0.44, 2.27)^∗^
Athlete (Subset)	0.95	15.37	(0.86, 1.04)^∗^
Item	0.00	0.00	N/A
Athlete × Subset	0.00	0.00	N/A
Item × Subset	0.00	0.00	N/A
Athlete × Stressor	3.21	51.90	(3.13, 3.29)^∗^
Stressor × Item	0.02	0.24	(0.00, 0.00)
Athlete × Item	0.05	0.81	(0.04, 0.06)^∗^
Error	0.61	9.78	(0.59, 0.62)^∗^
**Demands**			
Subset	0.00	0.00	N/A
Stressor (Subset)	0.50	20.07	(0.16, 0.84)^∗^
Athlete (Subset)	0.36	14.58	(0.33, 0.40)^∗^
Item	0.00	0.00	N/A
Athlete × Subset	0.00	0.00	N/A
Item × Subset	0.00	0.00	N/A
Athlete × Stressor	1.16	46.50	(1.13, 1.19)^∗^
Stressor × Item	0.01	0.53	(0.00, 0.00)
Athlete × Item	0.04	1.70	(0.04, 0.05)^∗^
Error	0.42	16.64	(0.40, 0.42)^∗^
**Resources**			
Subset	0.00	0.00	N/A
Stressor (Subset)	0.23	14.76	(0.07, 0.39)^∗^
Athlete (Subset)	0.31	19.94	(0.29, 0.34)^∗^
Item	0.00	0.00	N/A
Athlete × Subset	0.00	0.00	N/A
Item × Subset	0.00	0.00	N/A
Athlete × Stressor	0.83	52.63	(0.80, 0.85)^∗^
Stressor × Item	0.00	0.00	(0.00, 0.00)
Athlete × Item	0.01	0.50	(0.01, 0.01)^∗^
Error	0.19	12.20	(0.19, 0.20)^∗^

*Confidence intervals that do not cross zero indicate significant sources of variance (two-tailed p < 0.05). Components with a ^∗^ indicate significant sources of variance. Parentheses in the source column indicate that a component is nested within subsets.*

### Demand and Resource Evaluations

The percentages and significance of variance components for demand and resource evaluations, analyzed separately, are presented in Table 4. The Athlete × Stressor components accounted for the greatest amount of variance in both demand and resource evaluations (46.5 and 52.6%, respectively), suggesting that athletes had idiosyncrasies in their evaluations of how demanding the different stressors were, and their resources to cope with the stressors. These interaction components accounted for significantly greater amounts of variance in demand and resource evaluations than the Athlete (14.6 and 19.9%, respectively) and Stressor (20.1 and 14.8%, respectively) components, although these were also significant. The Athlete components imply that the athletes differed in how demanding they tended to view the stressors and their resources to cope with the stressors, regardless of the specific stressor characteristics. Conversely, the Stressor components suggest some agreement among the athletes in their tendency to view some stressors as more or less demanding than others, and that they had the resources to cope with some stressors better than others. The variance attributable to the Athlete and Stressor components did not differ significantly for either demand or resource evaluations.

## Discussion

Repeatedly viewing stressful situations as a threat (situational demands exceed coping resources) has been linked to negative health outcomes (e.g., depression; Blascovich, 2008b). However, it is not well-known if individuals have tendencies to evaluate all stressful situations as more of a challenge or threat (Power and Hill, 2010). Thus, this study aimed to shed light on this issue using generalizability theory (Cronbach et al., 1972). The generalizability analyses revealed differences between the athletes in their tendency to view stressors as more of a challenge or threat (athlete component), as well as some agreement among the athletes in their propensity to view some stressors as more of a challenge or threat than others (stressor component). Crucially, the results predominately indicated that athletes had idiosyncrasies in their tendency to view particular stressors as more of a challenge or threat (athlete × stressor interaction), with the interaction component explaining twice as much variance as each of the individual components. The same pattern of results emerged when demand and resource evaluations were analyzed separately.

The athlete component (person effect), or amount of variance in challenge and threat evaluations due to differences between athletes, was a significant, but also comparatively limited, source of variance. This result is congruent with previous research (e.g., Lucas et al., 2012), and has two contradictory implications. On one hand, the significant athlete component supports the notion that challenge and threat evaluations are, to some extent, relatively consistent across stressful situations, meaning that psychometric tools that assess individual differences in challenge and threat evaluations hold some merit (e.g., Tomaka et al., 2018). Indeed, such tools are likely to be useful for practitioners interested in selecting ‘challenge responders’ in high-pressure domains (e.g., medicine; Roberts et al., 2015), and researchers looking to monitor the long-term health of serial ‘threat responders’ (O’Donovan et al., 2012). On the other hand, the variance attributable to the athlete component was smaller than the other hypothesized components, which corroborates the often-cited concern that stress management interventions that overly rely on individual-based approaches, and ignore the specific environmental demands encountered by individuals, are less likely to be effective (only reflecting ‘damage limitation’), despite their ease of implementation and widespread popularity (Giga et al., 2003). Indeed, these approaches shift responsibility from the organization to the individual (Cooper et al., 2001), and would need careful consideration before being implemented in elite sport given the obligations that sport organizations have to safeguard athlete welfare [see UK Government’s, 2017; Duty of Care in Sport Review (Stevenson and Farmer, 2017)].

The stressor component (situation effect), or amount of variance in challenge and threat evaluations attributable to differences between stressors, was also a significant source of variance. Although sport- and sample-specific (i.e., female roller derby players), this result suggests that some stressors were more likely to be evaluated as a challenge (e.g., ‘high expectations’), while others were more likely to be seen as a threat (e.g., ‘negative coach behavior’), by all athletes. Interestingly, and in-keeping with prior research (e.g., Lucas et al., 2012), the stressor component was larger than the athlete component (albeit not significantly). This trend has implications for stress management interventions, and implies that interventions could be more effective if they focus on the environment rather than exclusively on the individual, an approach that is rarely adopted (Giga et al., 2003). For instance, armed with a list of stressors that athletes tend to evaluate as a threat (e.g., ‘inadequate preparation,’ ‘disruptive teammate’), practitioners could change the environment by altering particular features of these stressors (e.g., frequency, intensity, duration; Arnold and Fletcher, 2012b), or by removing the stressors altogether. However, despite such environment-based interventions being able to impact more athletes and having longer-lasting effects (Arnold et al., 2017), these interventions can be disruptive and difficult to implement logistically (Cooper, 2015), especially given that some stressors are uncontrollable from a practitioner’s perspective (e.g., ‘travel disruptions’). In the case of such unpredictable stressors, “what if” planning might prove a more viable strategy than trying to alter or remove the stressor (Karageorghis and Terry, 2011). Indeed, some researchers have argued against ‘sheltering’ athletes from stressors, instead noting the benefits associated with experiencing, and learning from, stressors (Collins et al., 2016).

The athlete × stressor interaction accounted for the greatest amount of variance in challenge and threat evaluations, significantly more than the athlete and stressor components, which is in-line with previous research that has reported large person × situation interaction effects for other psychosocial constructs (e.g., anxiety, social support; Lakey, 2016). Furthermore, this result offers direct support for transactional stress theories (e.g., cognitive appraisal theory; Lazarus and Folkman, 1984), which state that stress-related perceptions result from an exchange between the person and their environment. Indeed, the findings illustrate that whether a stressor is evaluated as a challenge or threat largely depends on who, specifically, is considering what particular stressor (Lucas et al., 2012). That is, athletes have different profiles of challenge and threat evaluations across the same stressors. For example, while one athlete might evaluate non-selection as more of a threat than an official’s poor decision, another might evaluate an official’s erroneous decision as more of a threat than non-selection. Although the significant athlete and stressor components imply that stress management interventions that focus solely on the individual or environment might be effective, the large interaction suggests that interventions are more likely to be beneficial if they adopt a conjoint approach (Giga et al., 2003). For instance, practitioners could equip athletes with individualized coping strategies that they can use when faced with the stressors that they find uniquely threatening (e.g., arousal reappraisal; Jamieson et al., 2018), while also working with sport organizations to alter or remove the stressors that each athlete finds particularly threatening (e.g., make selection process more objective, transparent, fair). Indeed, this approach would ensure that athletes and sport organizations are jointly responsible for managing stress (Rumbold et al., 2018).

Despite its novel methodology and important implications, this study has several limitations. First, the data was collected from a predominately female sample and single sport. While focusing on one sport helped create more specific vignettes that could be considered by all participants, it restricted the number of male participants and limited the generalizability of the results. Research suggests that females may be more likely to evaluate stressful situations as a threat than males (e.g., Quigley et al., 2002), thus, future research should test whether the sources of variance in challenge and threat revealed in this study hold for male-dominated samples and other sports. Second, some participants failed to fully complete the survey (∼17%), and therefore the results might have been influenced by non-completion bias (Mishra et al., 1993). Third, only self-report measures were used to assess challenge and threat. While this ensured data could be collected from a large athletic sample from all over the world, objective cardiovascular measures of challenge and threat are thought to be more accurate, unambiguous, and bias-free (Blascovich and Mendes, 2000; see Brimmell et al., 2018 for a recent application). Thus, future research should use generalizability analyses to uncover the consistency (or variability) of the cardiovascular markers of challenge and threat (total peripheral resistance and cardiac output reactivity), although this might be best achieved by asking participants to react to actual, rather than hypothetical, stressors. Indeed, the findings from such research could have important implications given that repeatedly reacting to stressful situations with a threat-like cardiovascular response has been linked to heart disease (Blascovich, 2008b).

To conclude, this study used generalizability theory to investigate the consistency (or variability) of challenge and threat evaluations across potentially stressful situations. The results revealed that the challenge and threat evaluations of athletes primarily comprised athlete × stressor interactions, suggesting that athletes had idiosyncrasies in their tendency to view certain stressors as a challenge or threat. The findings offer direct support for transactional stress theory, and imply that stress management interventions are more likely to be effective if they adopt a conjoint approach, equipping athletes with coping skills they can use when faced with the stressors they find particularly threatening, while simultaneously working with sport organizations to alter or remove these stressors.

## Data Availability

The raw data supporting the conclusions of this manuscript will be made available by the authors, without undue reservation, to any qualified researcher.

## Ethics Statement

This study was carried out in accordance with the code of ethics and conduct published by the British Psychological Society. The study protocol was approved by the Research Ethics Approval Committee for Health (REACH) at the University of Bath.

## Author Contributions

LM and ES-M led data collection, while PF and AH led data analysis. Finally, all authors contributed to the preparation of the study and writing of the manuscript.

## Conflict of Interest Statement

The authors declare that the research was conducted in the absence of any commercial or financial relationships that could be construed as a potential conflict of interest.
